# Regulatory Perspectives for Gambling in Brazil: are there Lessons that can be Learned from the UK´s Experience?

**DOI:** 10.1007/s10899-026-10496-1

**Published:** 2026-03-30

**Authors:** Eduardo Rocha Dias, Steve Greenfield

**Affiliations:** 1 Universidade de Fortaleza-UNIFOR, Avenida Washington Soares, Fortaleza, 1321, CEP 60811-905 CE Brazil; 2 University of Westminster, London, UK

**Keywords:** Gambling regulation, Gambling harms, Social determinants of gambling, Brazil, United Kingdom

## Abstract

Following recent enactment of online gambling regulations in Brazil, this commentary aims to compare the country´s policies with those of the UK, where the Gambling Act has been in force since 2005. Brazil followed a trend that falls within the recent global expansion of the gambling industry, although having an internal dynamic of its own The key question for those jurisdictions exploring deregulation or who have already ventured down that path is how a regulatory framework can reconcile the ‘harm’ that accompanies the increased freedom to gamble alongside the economic benefits that choice generates. This is a clear obstacle to framing policy in public health terms as opposed to one based on market freedom. The ongoing experience of Brazil demonstrates that, despite the wealth of evidence relating to gambling harms from the UK, the embracing of a neo-liberal approach still resonates. Regulators in Brazil and beyond need to be critically aware of the resistance to introducing new restrictions, from the gambling companies themselves, and respond accordingly.

In 2025, Brazil began applying regulations issued following legalization of online gambling in the country. That came roughly 20 years after the UK enacted the Gambling Act 2005, that has witnessed a huge expansion in gambling and associated problems (House of Lords, 2020). This commentary aims to compare Brazil´s policies with those of the UK, which is pursuing some limited reforms (United Kingdom, 2023) to mitigate gambling-related harms; yet the fundamental problem of balancing the different interests remains. As the House of Lords noted:

‘The much publicised position of the gambling industry is that the great majority of gamblers gamble within their means and spend no more on gambling than they would on any other activity which gives them enjoyment. It adds zest to their lives, and particularly to their enjoyment of sport. They should be free to do so. Added to which, the industry stresses the benefits to society, to employment, to tourism and to taxation.’ (House of Lords, 2020, 9).

The key question for those jurisdictions exploring deregulation or who have already ventured down that path is how a regulatory framework can reconcile the ‘harm’ that accompanies the increased freedom to gamble alongside the economic benefits that choice generates. Governments are inevitably attracted by the economic benefits even if other indirect ‘costs’ such as healthcare are hidden.

The UK has underpinned gambling deregulation with a neo-liberal, market and business-oriented approach, favoring competition, consumer choice and minimal state intervention (Reith & Wardle, 2022). One of the drivers of this strategy is the global nature of the gambling industry with a concomitant fear of losing markets if regulation is perceived, by consumers, as too rigid. Opponents to deregulation frame the argument in terms of public health by focusing on the associated harms (Orford, 2020).

Brazil followed a trend that falls within the recent global expansion of the gambling industry (Wardle et al., 2024), although having an internal dynamic of its own. Regulation has followed a sinuous and sometimes contradictory path (Jobim, 2022, p. 273). In 2018, Law 13,756 (LEI Nº 13.756/2018, 2018) legalized fixed-odd bets, that is, related to real events in which it is defined, at the time the bet is placed, how much the bettor can win if the prediction is correct. No further regulations were issued until 2023, when Law 14,790 (LEI Nº 14.790/2023, 2023) included among fixed-odd bets those related to virtual events (online casinos), also to be exploited by private companies on a license-based scheme. The country´s national regulator was created in 2024, the Secretariat of Prizes and Betting (SPA), a branch of the Ministry of Finance, which hints that the main concerns were related to tax collection. It was only in 2025 that the regulations issued by SPA became effective.

The most recent SPA Report (Brazil, 2026) indicates the volume of betting taking place, almost 12% of the population, or 25.2 million people, placed a bet of sorts. 79 companies were licensed with gross gambling revenue (GGR) in 2025 of R$36.9 billion (£5.2 billion). In 2025, tax revenues reached R$9.95 billion, while licensing fees generated an additional R$2.5 billion. These figures highlight the fiscal appeal of deregulation. The Ministry of Sports and sports committees are the main beneficiaries, receiving 36% of the revenues, whereas the Ministry of Health receives only 1%.

There are restrictions to advertising and marketing, aiming at protecting those below 18 years of age. Advertising must observe regulations issued by SPA and self-regulation is also incentivized. However, the restrictions do not match those relating to alcoholic beverages with alcoholic content above 13 degrees Gay-Lussac and tobacco products which are not allowed to sponsor sports activities for instance. This contradicts evidence indicating that 7.3% of the Brazilian population aged over 14 - approximately 10.9 million people – engage in gambling in ways that cause financial, emotional, familial, or work-related harm (Unifesp, 2025, p. 40) and it violates the Brazilian Constitution, which imposes on the State a duty to protect families from harmful products and activities. According to the latest National Survey on Drugs and Alcohol (LENAD III), conducted between 2023 and 2024, 2.6% of individuals are at moderate risk of gambling addiction and 0.8% at high risk on the PGSI scale (Unifesp, 2025, p. 40). Overall, 82.6% report not gambling, 6% fewer than in the first survey conducted in 2005–2006 (Tavares et al., 2010).

Each operator must define its responsible gambling policy and monitor gamblers accordingly. Time and expenditure limit setting, warnings, self-exclusion and other control mechanisms must be included in such policy. Analytical tools and classification and data analysis methodology must also be used to monitor and assess a gambler´s risk profile for pathological gambling and other associated problems. However, each operator’s policy defines how gamblers are monitored, and the Brazilian regulator has not established risk indicators that must trigger protective measures. In the UK, the Gambling Commission has defined a set of harm indicators that operators are expected to monitor (United Kingdom, 2023, pp. 31–32).

A 2024 report by the Brazilian Central Bank estimates that, from January to August 2024, Brazilians transferred between R$18 and R$21 billion (£2.6–£3 billion) per month to gambling operators through the PIX system alone (Brazil 2024a). The consumer expenditure in the gambling sector has impacted on other retail revenue. This led the National Confederation of Commerce of Goods, Services and Tourism filing a constitutional complaint before Brazil´s Supreme Court (Brazil 2024b), challenging the constitutionality of Law 14,790/2023. The court has not yet rendered a final decision, which may affect future developments concerning gambling regulation in Brazil, considering that draft bills that legalize land-based casinos and bingos are now under analysis by the country´s parliament. A court order was issued determining that the Government should adopt measures to prevent money from income transfer programs, like the Bolsa-Família program, from being used in gambling. This was viewed by part of society as a legitimate attempt to protect the most vulnerable, although others deemed it paternalistic. However, the feasibility of the order is questionable, since there appears to be no way to distinguish funds originating from the program from those derived from other income sources once they are transferred. It was noted by the Brazilian Federal Court of Accounts that some beneficiaries of the program spent unexplainably high amounts on gambling, which is a worrying indicator that they are being used for money laundering or fraud (Brazil, 2025). This discussion is an opportunity to consider how to protect the most vulnerable from harm arising from gambling through the assessment of their financial transactions. UK Open Banking laws, in this regard, may provide an interesting tool to assess in an objective way, in accordance with a public health approach, individuals´ behavior regarding gambling and the harm they are subjected to (Ghaharian et al., 2025; Murphy & Muggleton, 2025).

A key feature in both the UK and Brazil is the integration of gambling into the professional football sector, thereby normalizing this relationship despite the contradiction between sport and activities that generate harm. In 2025 the value of sponsorship contracts with football teams reached R$1.117 billion (£ 160 m), 125% higher than in the 2023 season, when it was of R$496 million (£ 71 m) (Catto, 2025). All teams of the country´s main division, A Series, during the 2025 season, were sponsored by gambling companies, with 18 as a main sponsor, with their logos visible on an important part of shirts and uniforms. By creating financial dependency, the Brazilian football sector consequently has a vested interest in limiting restrictions and promoting expansion (Marionneau et al., 2022, p. 2). In the UK one of the reforms has been a voluntary restriction by football clubs on shirt advertising, as a response to public criticism, though the likely overall impact is dubious given the other advertising opportunities that still exist. This approach is not mirrored in Brazil: the day before the Brazilian Senate´s Sport Commission met to discuss the draft bill 2,985/2023, which aimed to restrict advertising of gambling companies, a joint declaration of dozens of clubs was published expressing their concern and the risk of “collapse” of Brazilian football if such restrictions became legally binding (Gonçalves, 2025).

There are opportunities to influence regulations issued by the SPA by participating in public consultations on its regulatory agenda. It is also worth noting the creation, in 2024, of a working group bringing together representatives from the Ministries of Finance, Sports, and Health, which may lead to positive measures. At the end of 2025, because of this group’s activities, a centralized self-exclusion mechanism similar to the British GAMSTOP was introduced (Brazil, 2026). The group will also work to strengthen the country’s health system to better address the negative consequences of gambling and may draw lessons from how the NHS tackles the issue.

## Conclusion

The main lesson Brazil may draw from the UK experience is that framing gambling regulation according to a public health approach is difficult and faces resistance not only from the gambling industry, but also for those who benefit from the income generated by the activity. Another lesson is that organized pressure from society can produce positive changes, as demonstrated by the removal of gambling companies’ logos from shirts by the Premier League, even if the impact of such measures remains limited, given the persistence of other forms of messaging and advertising. The adoption of a centralized self-exclusion mechanism in Brazil and the introduction of specialized care in the country´s healthcare system are also clearly inspired by similar measures found in the UK and resulted from contributions from health professionals. Further restrictions on advertising, sports sponsorship, and other social determinants of gambling are likely to remain minimal, as shown by the limited progress in Brazil’s Parliament due to pressure from the sports sector. The same may apply to the constitutionality challenge of Law 14,790/2023 before Brazil’s Supreme Court, despite its clear inconsistency with harm-reduction policies on alcohol and tobacco. To enhance protection against gambling-related harm, Brazilian regulation could require operators to monitor harm indicators - similar to the way the UK Gambling Commission does - and impose stricter advertising limits, such as banning sign-up offers on TV before certain hours, while expanding consumer choice through opt-in controls over marketing content and channels, a measure that came into force in the UK in May 2025 (Woodhouse, 2026, pp. 10–11). Placing the regulator (SPA) under the Ministry of Health as an independent agency would also benefit harm reduction. In the UK, where the Gambling Commission sits within the Department for Culture, Media and Sport, there are calls to move it to the Department of Health and Social Care (Reith & Wardle, 2022).

The ongoing experience of Brazil demonstrates that, despite the wealth of evidence relating to gambling harms from the UK, the attraction of a neo-liberal approach still resonates. Thus, regardless of the underlying political philosophy, regulators in Brazil and elsewhere must remain aware of the resistance to introducing new restrictions, from gambling companies themselves and those with vested interests in the activity and respond accordingly. Effectively balancing the different interests, as the House of Lords noted at the outset (House of Lords, 2020), is a common and increasingly difficult proposition.
